# Clinical practice of image-guided spine radiosurgery - results from an international research consortium

**DOI:** 10.1186/1748-717X-6-172

**Published:** 2011-12-15

**Authors:** Matthias Guckenberger, Reinhart A Sweeney, John C Flickinger, Peter C Gerszten, Ronald Kersh, Jason Sheehan, Arjun Sahgal

**Affiliations:** 1 Department of Radiation Oncology, University Hospital Wuerzburg, Wuerzburg, Germany; 2 Department Radiation Oncology, University of Pittsburgh School of Medicine, Pittsburgh, Pennsylvania, USA; 3 Center for Image-Guided Neurosurgery, University of Pittsburgh School of Medicine, Pittsburgh, Pennsylvania, USA; 4 Department of Neurological Surgery, University of Pittsburgh School of Medicine, Pittsburgh, Pennsylvania, USA; 5 Department of Radiation Oncology, University of Virginia, Charlottesville, Virginia, USA; 6 Riverside Medical Center, Newport News, Virginia, USA; 7 Department of Neurological Surgery, University of Virginia School of Medicine, Charlottesville, Virginia, USA; 8 Dept. of Radiation Oncology, Princess Margaret Hospital, Toronto, Ontario, Canada

**Keywords:** vertebral metastases, spine radiosurgery, methods, questionnaire

## Abstract

**Background:**

Spinal radiosurgery is a quickly evolving technique in the radiotherapy and neurosurgical communities. However, the methods of spine radiosurgery have not been standardized. This article describes the results of a survey about the methods of spine radiosurgery at five international institutions.

**Methods:**

All institutions are members of the Elekta Spine Radiosurgery Research Consortium and have a dedicated research and clinical focus on image-guided radiosurgery. The questionnaire consisted of 75 items covering all major steps of spine radiosurgery.

**Results:**

Strong agreement in the methods of spine radiosurgery was observed. In particular, similarities were observed with safety and quality assurance playing an important role in the methods of all institutions, cooperation between neurosurgeons and radiation oncologists in case selection, dedicated imaging for target- and organ-at-risk delineation, application of proper safety margins for the target volume and organs-at-risk, conformal planning and precise image-guided treatment delivery, and close clinical and radiological follow-up. In contrast, three major areas of uncertainty and disagreement were identified: 1) Indications and contra-indications for spine radiosurgery; 2) treatment dose and fractionation and 3) tolerance dose of the spinal cord.

**Conclusions:**

Results of this study reflect the current practice of spine radiosurgery in large academic centers. Despite close agreement was observed in many steps of spine radiosurgery, further research in form of retrospective and especially prospective studies is required to refine the details of spinal radiosurgery in terms of safety and efficacy.

## Background

Radiotherapy is a well-established treatment for painful vertebral metastases. Multiple prospective studies report pain response rates of 50 to 90% [1-4]. Based on randomized studies, no differences in pain response have been observed between the various fractionation schemes that range from 40 Gy in 20 fractions to 8 Gy in a single fraction [5,6].

Despite the lack of a dose response relationship for pain control, there is good rationale for high dose escalation beyond those conventional dose levels tested with the aim to improve upon existing rates of local and pain control. The median duration of pain response after conventional palliative radiotherapy is approximately 3 to 6 months, again without differences between the different fractionation schemes [1-4]. Only short palliation after conventional radiotherapy seems to be the case especially for unfavorable histologies (lung, kidney, head & neck and gastrointestinal cancer, melanoma, sarcoma) as observed in a randomized trial for metastatic spinal cord compression [7]. This brief palliative effect may be sufficient for some patients with very short life expectancy. However, several predictive scores have been reported, which allow selection of patients with long life expectancy [8-10]. In particular, as modern chemotherapy may further prolong life expectancy, long-term palliation and long-term tumor control become even more important goals for patients despite having metastatic disease.

As a consequence, there is currently large interest in intensification of radiotherapy for painful vertebral metastases. A recent survey from the Unites States reported that 64% of the radiation oncologists practice stereotactic body radiotherapy (SBRT) and treatment of vertebral metastases was the second most common disease site (67.5% of all SBRT users) [11]. Spine SBRT was practiced most frequently as single fraction radiosurgery with doses of 20 Gy or 18 Gy. The most frequently cited reasons for practice of SBRT were the possibility of dose-intensified treatment and re-irradiation. In the remainder of this manuscript, we will use the term radiosurgery for both single-fraction and multiple-fraction SBRT as the techniques of the entire treatment processes are identical between both.

Multiple retrospective and few prospective studies have reported promising results for spine radiosurgery with low rates of toxicity and pain control as well as local tumor control rates consistently ranging between 70 - 90% [12-18]. Importantly, the risk of permanent spinal cord damage secondary to radiation induced myelopathy has been reported as very low. Despite these promising data, recent reviews have pointed out the lack of uniformity in practice [19-21], and currently the community has no standard approach to the practice of spine radiosurgery. For example, there is variability in the treatment techniques, the total dose prescribed to the tumor, number of fractions, criteria for plan acceptance and the dose limits used to the organs at risk. One recent advance in providing guidance and uniformity to the practice of spine radiosurgery has been the RTOG phase II/III trial that is currently accruing in the US (RTOG 0631); however, results of this trial will not be available in the near future.

The purpose of our study is to give a broad and comprehensive overview of the current methods of spine radiosurgery by surveying experienced practitioners. Five institutions, all members of the international Elekta Spine Radiosurgery Research Consortium, answered a questionnaire with detailed questions about all steps of spine radiosurgery from indication to follow-up. All centers used identical equipment for treatment delivery (Elekta Synergy S linacs all equipped with cone-beam and robotic HexaPOD technology), which facilitates methods comparison between the institutions.

## Methods

The Elekta Spine Radiosurgery Research Consortium (ESRRC) is an international research consortium consisting of five institutions, all of them with a research and clinical focus on image-guided high precision radiotherapy in general and spine radiosurgery in particular. Four of five institutions are academic hospitals (University Hospital Wuerzburg [UHW], Wuerzburg, Germany; Princess Margaret Hospital (PMH) and the Sunnybrook Health Sciences Center (SHSC) of the University of Toronto [UofT], Toronto, Canada; University of Pittsburgh Medical Center [UPMC], Pittsburgh, US; University of Virginia Medical Center [UVAMC], Charlottesville, US) and one is a private radiotherapy center fully specialized in image-guided radiosurgery (Riverside Regional Medical Center [RSMC], Newport News, US). All institutions have treated more than 50 patients with vertebral metastases using image-guided radiosurgery and all academic centers have contributed to the recent technical and clinical progress in spine SBRT.

Each center uses identical equipment for delivery of spine radiosurgery: treatment is planned for a high-resolution multi-leaf collimator with 4 mm leaf width (Beam modulator on Elekta Synergy S linear accelerator; Elekta, Crawley, UK), daily volumetric image-guidance is performed with cone-beam technology (Elekta XVI, Crawley, UK), set-up errors are corrected in six degrees of freedom (HexaPOD; Medical Intelligence, Schwabmuenchen) and all patients are immobilized in the BodyFIX system (Medical Intelligence, Schwabmuenchen, Germany).

A questionnaire with 75 items was established covering all major aspects of spine radiosurgery including: indications for spine radiosurgery, imaging required for treatment planning, target and organ-at-risk (OAR) definition, treatment planning, dose and fractionation, tolerance doses for OARs, patient positioning and image-guidance, follow-up and response evaluation. Re-irradiation and post-operative radiosurgery was not evaluated in this questionnaire. The questionnaires were answered by the responsible physician from each institution and reflect their current practice of spinal radiosurgery.

## Results

### Indication for spine radiosurgery

Rationales for the practice of spine radiosurgery compared to conventional palliative radiotherapy are similar between the five institutions: all agree on more durable pain control and long-term local tumor control. Four institutions state a more rapid pain relief as reason for spine radiosurgery (UPMC, UofT, UVAMC, RSMC), three institutions explicitly mention spine radiosurgery for radio-resistant histologies (UHW, UVAMC, UofT), two institutions use spine radiosurgery because of better patient convenience (UPMC, RSMC) and one institution describes the potential of improved overall survival in the oligometastatic setting as reason for spine radiosurgery (UHW). Radiosensitive histologies are excluded in all but one (RSMC) institution and a minimum performance status is required in all institutions (table 1). All four academic centers perform spine radiosurgery in the framework of a prospective protocol but not as a prospective trial.

**Table 1:** Patient specific factors influencing indication for spine SBRT

	UHW	UPMC	UofT	UVAMC	RSMC
Use of a predictive scoring system for OS	Yes, Mizumoto Score	No	Life expectancy ≥ 3 months	Patients with widespread CNS and systemic disease are excluded	No

Histology of primary tumor	No treatment of highly radiosensitive histologies	Avoid relatively radiosensitive histologies	No myeloma unless previously radiated	No treatment of radiosensitive histologies	No relevant factor

Status of primary tumor	Yes, part of the Mizumoto Score	No relevant factor	Yes, for estimation of life expectancy	No relevant factor	No relevant factor

Presence of visceral metastases	Yes, part of the Mizumoto Score	No relevant factor	Yes, for estimation of life expectancy	Yes, see above	No relevant factor

Age	Yes, part of the Mizumoto Score	No relevant factor	No relevant factor	No relevant factor	No relevant factor

Performance status of patient	Yes, part of the Mizumoto Score	Exclusion of patients with extremely poor performance status	Must be able to tolerate immobilization for 45 min.	KPS should be ≥70	KPS must be ≥60

Comorbidities of patient	No relevant factor	No relevant factor	No relevant factor	No relevant factor	No relevant factor

Interval between primary tumor and spinal metastases	No relevant factor	No relevant factor	No relevant factor	No relevant factor	No relevant factor

In contrast, patient selection with respect to estimated live expectancy is substantially different: one institution strictly selects patients with good life expectancy using a predictive scoring system for overall survival (UHW), two centers exclude patients with very poor life expectancy (UofT, UVAMC) and life expectancy is no relevant factor for two institutions (UPMC, RSMC).

### Characteristics of metastatic lesions treated with spine radiosurgery

The inclusion and exclusion criteria of vertebral metastasis treated with spine radiosurgery are described in table 2. There is agreement that the relationship between the target volume and any OAR other than the spinal cord does not influence the indication for spine radiosurgery. Both lytic and sclerotic lesions are treated and all institutions but one (UHW) prefer a stabilization procedure prior to radiosurgery in cases of spinal instability. Compression fractures are always discussed with the neurosurgeon/spine surgeon and symptomatic spinal cord compression is a contraindication in all institutions. All vertebras in the cervical, thoracic and lumbar spine are treated and the number of vertebras within one target volume is limited to 3 except one institution, were only two vertebras are allowed within one target volume (RSMC).

**Table 2:** Target specific factors influencing indication for spine SBRT

	UHW	UPMC	UofT	UVAMC	RSMC
Location of vertebral metastases (C, T, L)	No relevant factor	No relevant factor	No relevant factor	No relevant factor	No relevant factor

Number of vertebras in one target volume	Maximum of 3 levels	Maximum of 3 levels	Maximum of 3 levels	Maximum of 3 levels	Maximum of 2 levels

Extent of vertebral metastases	Symptomatic and progressive cord compression is contraindication.	Significant spinal cord compression associated with myelopathy is contraindication.	Symptomatic cord compression is contraindication.	Symptomatic cord compression is contraindication.	Symptomatic cord compression is contraindication.

Epidural involvement	No relevant factor	No relevant factor	Surgery if high grade epidural involvement present	Minimum of 2 mm of clearance between the gross metastastic disease and the spinal cord	No relevant factor

Stability of metastatic vertebra	Surgical opinion sought first	Instability is preferably treated with stabilization procedure	Surgical opinion sought first	Instability is preferably treated with stabilization procedure	Instability is preferably treated with stabilization procedure

Lytic or sclerotic metastasis	No relevant factor	No relevant factor	No relevant factor	No relevant factor	No relevant factor

Vertebral compression fracture	Symptomatic compression fracture are discussed with neurosurgeons in advance	Compression fracture causing kyphosis and pain will be treated BEFORE radiosurgery if possible	Surgical opinion sought first	Compression fracture causing marked kyphosis or instability will be treated with stabilization procedure first	Compression fracture is preferably treated with stabilization procedure

Location of metastasis relative to other organs at risk	No relevant factor	No relevant factor	No relevant factor	No relevant factor	No relevant factor

Disagreement is evident whether epidural involvement or a small distance between the metastasis and the spinal cord are contraindications.

### Imaging for staging and target definition

There is a good agreement in the imaging modalities and their technical application for staging and target definition (table 3). All institutions acquire dedicated CT and MRI images (a diagnostic MRI is allowed at the UPMC) for delineation and slice thickness is between 1-2 mm.

**Table 3:** Imaging for staging & target definition

	UHW	UPMC	UofT	UVAMC	RSMC
Staging examinations prior to SBRT/SRS	Oncologic staging is required	None	MRI spine	None	None

Slice thickness of Planning CT	1.5 mm	1.25 mm	1 mm	1 to 1.5 mm	2 mm

MRI used for target definition	Yes	Yes	Yes	Yes	Yes

Dedicated Planning MRI	Yes	No	Yes	Yes	Yes

Slice thickness of planning MRI	2 mm	1.25 mm	1 mm	1.2 mm	3 mm/1.25 mm

MRI sequence used for target definition,	T1 with and w/o contrast; T2	T1 with contrast; T2	T1 w/o contrast volumetric VIBE; T2 volumetric SPACE	T1 with contrast volume acquisition	T1 with and w/o

Dedicated FDG-PET/PET-CT for target definition	Rarely	Yes	No	Rarely	Yes

Differences, however, are observed in the MRI sequences and in acquisition of a dedicated planning FDG-PET.

### Target and OAR definition

Similar target volume concepts are used in the five institutions (table 4). All centers define the gross-tumor volume (GTV) based on CT and MR imaging, two centers perform co-registration of a FDG-PET (UPMC, RSMC). All centers treat the involved vertebras only without "prophylactic" irradiation of the superior and inferior vertebra. All institutions use an anatomical target volume concept where the target volume extends to uninvolved parts of the vertebras. Additionally, all institutions but one (RSMC) apply safety margins of 2-3 mm.

**Table 4:** Target and organs-at-risk definition

	UHW	UPMC	UofT	UVAMC	RSMC
Imaging modality, which is used for GTV definition	MRI and CT	MRI and CT, FDG-PET if available	MRI and CT	CT and MRI	CT, MRI and FDG-PET

Use of an anatomical target volume concept	Anatomical two dose-level target volume concept	Anatomical target volume concept	Anatomical target volume concept	Anatomical target volume concept	Anatomical target volume concept

GTV to PTV safety margin	3 mm	2 mm; 3 mm in the sacrum.	2 mm	2 mm	None

Protocol if PTV overlaps with the. spinal cord	Two dose-level approach; The OAR spinal cord is always in the PTV-elective and is always excluded from the higher dose PTV-macroscopic	PTV within 1 mm to the spinal cord is excluded from the PTV	PTV is limited by the cord or thecal sac for cauda equina	If this occurs, we either operate to resect part of the tumor or fractionate the radiation.	GTV drawn to edge of OAR

Treatment of the vertebra superior and inferior to the metastatic vertebra	No	No	No	No	No

Imaging modality for definition of the spinal cord	Spinal cord in MRI	Spinal cord in MRI	Spinal cord in MRI	Spinal cord in MRI	Spinal canal in CT

Delineation of the spinal cord in cranio-caudal direction	At least 1 level above and below PTV	1 level above and below PTV	At least 1 level above and below PTV	1 level above and below PTV	1 level above and below PTV

Safety margins around the spinal cord in axial directions	1 mm	1 mm	1.5 mm	No	2 mm anterior and 1 mm lateral

Delineation of the cauda equina	Thecal sac	Thecal sac	Thecal sac	Thecal sac	Thecal sac

Delineation other OARs (e.g. kidney)	No application of safety margins	No application of safety margins	No application of safety margins	No application of safety margins	No application of safety margins

However, the details of the target volume concepts are different. Three institutions do always treated the entire vertebral body and/or the entire posterior elements in case of involvement (UPMC, UVAMC, RSMC). One institution has a similar concept, however, differentiates between the ipsilateral and contralateral posterior elements (UofT). One institution uses a two dose level approach where the high-dose target volume is defined as the GTV with a 3 mm safety margin and the low-dose target volume is the entire vertebra (UHW).

Regarding definition of the OAR spinal cord, all but one institution (RSMC) define the spinal cord in the MRI images; the spinal canal is delineated in CT images at the RSMC. Delineation is performed minimum 1 vertebra superior and inferior to the planning target volume (PTV) in all institutions and safety margins of 1-2 mm are applied for generation of the planning OAR spinal cord in all but one institution (UVAMC). On the level of the cauda equina, all institutions define the thecal sac as planning OAR.

### Treatment dose and fractionation

Large variability is observed in terms of treatment dose and fractionation (tables 5). Two institutions treat the majority of their patients with single fraction radiosurgery of 16 - 24 Gy (UPMC, UVAMC). One center prefers 2 or 3 fraction radiosurgery (UofT), however, will treat with single fraction if no epidural disease is evident and single level disease. Two centers perform fractionated radiosurgery only (UHW, RSMC), and both of them choose between two fractionation schemas with estimated life expectancy as selection criterion. Fractionated radiosurgery is performed in 2-10 fractions with physical doses of 24 - 48.5 Gy. Despite the differences in dose and fractionation, all but one institution practice dose prescription to the D90, whereas one institution uses the ICRU reference point (UofT).

**Table 5:** Doses and fractionation

	UHW	UPMC	UofT	UVAMC	RSMC
Use of single fraction radiosurgery	No, all patients are treated with either five or ten fractions	Single fraction radiosurgery for 95% of the patients unless very near to spinal cord.	Majority is treated with two or three fractions and specific cases for single fraction	Majority is treated with a single fraction of radiosurgery, occasionally up to 3 fractions	No, majority are treated with three fractions with treatments given one week apart.

Criteria for selection of hypo-fractionated regimes	Selection of fractionation scheme based on life expectancy using the Mizumoto Score		Fractionated protocols in: 1. Epidural disease or large volume and no prior irradiation 2. Prior radiation	Fractionated protocols after prior radiation	If it represents the only site of disease, we use 30 Gy in 3

Schema 1: # fractions and single fraction dose	Good life expectancy: 30 Gy in 10: PTV-elective 48.5 Gy in 10: PTV -macroscopic *	16-24 Gy in 1; Most frequently 17 Gy in 1	20-24 Gy in 1; Most frequently 20 Gy in 1	18 to 24 Gy in 1; Most frequently 20 Gy in 1	24 Gy in 3

Schema 2: # fractions and single fraction dose	Intermediate life expectancy: 20 Gy in 5: PTV-elective 35 Gy in 5: PTV -macroscopic *		24 - 27 Gy in 2-3	24 Gy in 3	30 Gy in 3

Schema 3: # fractions and single fraction dose			30 Gy in 3 (for sarcomas)	18 Gy in 3	

Dose prescription	D90	D90	ICRU point	D90	D90

* a simultaneous integrated boost (SIB) was used at the UHW with two dose levels to PTV -macroscopic and PTV -elective

Based on an α/β = 10 Gy, the median 2 Gy-equivalent dose (EQD_2 Gy_) is 50 Gy [minimum 36 Gy (3 × 8 Gy) and maximum 68 Gy (1 × 24 Gy)]. As described above, one institution (UHW) uses a two dose-level concept with conventional doses in the "elective" parts of the vertebra (10 × 3 Gy; 5 × 4 Gy) and dose escalated irradiation in the involved parts of the vertebra (10 × 4.85 Gy; 5 × 7 Gy).

### Spinal cord tolerance doses

No institution varies the spinal cord tolerance based on cervical, thoracic or lumbar target location. Otherwise, dosimetric parameters as well as tolerance doses for the spinal cord and thecal sac were substantially different between all five institutions (table 6).

**Table 6:** Spinal cord tolerance doses

		Tolerance doses Spinal Cord			
	**Dosimetric parameter**	**Single fraction**	**3 fractions**	**5 fractions**	**10 fractions**

UHW	Dmax to 0.1 cc			23.75 Gy	35 Gy

UPMC	Dmax	11 Gy	18 Gy		

UofT	Dmax	10 Gy	17.5 Gy	22 Gy	

UVAMC	D10	10 Gy	15 Gy	20 Gy	

RSMC	2 cc		18 Gy		

		**Tolerance doses Cauda equina**			

	**Dosimetric parameter**	**Single fraction**	**3 fractions**	**5 fractions**	**10 fractions**

UHW	Dmax to 0.1 cc			25 Gy	37.5 Gy

UPMC	Dmax	12 Gy	18 Gy		

UofT	Dmax	12 Gy	18 Gy	23 Gy	

UVAMC	D10	12 Gy	15 Gy	20 Gy	

RSMC	2 cc		24 Gy		

### Treatment planning

Minor differences are observed in terms of treatment planning (table 7). All institutions treat their patients at an Elekta Synergy S linear accelerator equipped with the Beam Modulator (4 mm leaf width); one center does also perform spine radiosurgery on different linear accelerators (UVAMC). Treatment planning system is Pinnacle (Philips Radiation Oncology Systems, Milpitas, CA, USA) and intensity modulation is planed using step-and-shoot IMRT only (UPMC), both IMRT and VMAT (UHW, RSMC, UofT) and VMAT only (UVAMC). Technical details are summarized in table 7.

**Table 7:** Treatment planning

	UHW	UPMC	UofT	UVAMC	RSMC
Treatment planning system	Pinnacle	Pinnacle	Pinnacle	Varian Eclipse, Tomotherapy, Pinnacle for Elekta	1. Elekta/CMS XiO, 2. Elekta/CMS Monaco

Linac model/MLC leaf width	Elekta Synergy S/4 mm	Synergy S/4 mm	Elekta Synergy S/4 mm	Elekta Synergy S, Varian Triliogy, Tomotherapy	Elekta Synergy S/4 mm

IMRT or VMAT treatment planning	Both	IMRT	Both	VMAT	Both

If step-and-shoot IMRT: number of beams	9 beams on average	9 to 14, but most are 12 beams	9 - 11 beams	10 beams	10 beams

If VMAT: number of arcs	1-2 arcs	Not applicable	1 arc	1-3 arcs	1. one arc 120-140 segments 2. VMAT - 4 arcs - 30-40 segments per arc

Full or partial VMAT arc	360 degrees	360 degrees	360 degrees	360 degree arcs	1. VMAT 350 deg arc 2. VMAT posteriorly biased arcs, 2 couch kicks

Photon energy	6 or 10 mV depending on location	6 MV	6 MV	6 MV	6 MV

Dosimetric parameters for plan acceptance	No strict acceptance criteria.	Usually V90	CTV V80 of at least 80-90%	No strict acceptance criteria	Generally D90

Acceptance criteria for treatment plans vary substantially with all centers stating that no strict criteria exist, mostly because of the large variability of the target volumes in terms of size, shape and distance to the spinal cord and other relevant OARs. However, all institutions agree that PTV coverage is sacrificed until the dose limits of the critical OARs, especially of the spinal cord, are fulfilled.

### Patient positioning, immobilization and image-guidance

Differences in these steps of spine radiosurgery are small (table 8). All patients are treated in supine position and immobilization is performed using thermoplastic head masks for cervical/upper thoracic lesions and the BodyFIX for thoracic and lumbar lesions. Daily pre-treatment image guidance is performed using cone-beam technology and set-up errors are corrected in six degrees of freedom using the robotic HexaPOD couch. Action level for translational errors is 1 mm in all but one institution where a larger action level of 2 mm is used (UVAMC); the action level for rotational errors is 1° in four institutions (UHW, UPMC, UofT, UVAMC), 0.3° in one institution (RSMC). A second cone-beam CT scan for verification of the IGRT shift is performed in all institutions and all institutions but one (UHW) perform intra-treatment cone-beam CT scanning for patient monitoring. A final scan after treatment delivery is performed in 3/5 institutions (UHW, UofT, UVAMC).

**Table 8:** Patient (re-)positioning and IGRT

	UHW	UPMC	UofT	UVAMC	RSMC
Treatment prone or supine	Supine	Supine	Supine	supine	Supine

Immobilization device	Cervical: Thermoplastic mask; otherwise BodyFIX	Cervical down to T5: aquaplast face mask; otherwise BodyFIX	Cervical down to T2/3 s frame; otherwise BodyFIX	Cervical: Thermoplastic mask; otherwise BodyFIX	Cervical: Aquaplast mask with Accuform support secured to modified S-frame; otherwise BodyFIX

Image guidance technology	Cone-beam CT	Cone-beam CT	Cone-beam CT	Cone-beam CT	Cone-beam CT

Frequency of IGRT	Daily	Daily	Daily	Daily	Daily

Correction of rotational set-up errors	Yes - Hexapod couch	Yes - Hexapod couch	Yes - Hexapod couch	Yes - Hexapod couch	Yes - Hexapod couch

Action level for correction of set-up errors	1 mm translation, 1 degree rotation	1 mm translation, 1 degree rotation	1 mm translation, 1 degree rotation	2 mm translation, 1 degree rotation	1 mm translation, 0.3 degree rotation

Second imaging after couch adjustment prior to treatment	Yes	Yes	Yes	Yes	Yes

Methods for intra-fractional patient monitoring	None	Cone-beam CT imaging after one and two thirds through the treatment	One to two intra-treatment Cone-beam CT scans	One intra-fraction cone-beam CT scan half-way through treatment	Typical one or two mid treatment cone-beam CTs

Imaging after treatment	Yes	No	Yes	Yes	No

### Follow-up

Follow up is performed in-house whenever possible in all institutions; the interval is most frequently every 3 months (table 9). Local tumor control is defined as tumor shrinkage or no tumor progression in serial imaging, with MRI as the preferable imaging modality. One institution (RSMC) uses routine FDG-PET imaging for evaluation of local tumor control. Pain is assessed in 4/5 institutions using either the Visual Analog Scale (UHW, UPMC, UVAMC) or NRS-11 (RSMC).

**Table 9:** Follow-up and response evaluation

	UHW	UPMC	UofT	UVAMC	RSMC
Place of follow-up	In clinic	In clinic	In clinic	In clinic	In clinic

Definition of local control	No progression on serial imaging.	No progression on serial imaging.	No progression on serial imaging.	No progression on serial imaging.	No progression on serial imaging.

Imaging modalities required for definition of local control	MRI if possible	MRI if possible	MRI if possible	MRI if possible	MRI/PET

System for pain scoring	Visual analogue scale	Visual analogue scale	N/A unless of study then the Brief Pain Inventory	Visual analogue scale	NRS-11

Frequency of FU examinations	Every three months, every six months after 1 year	1 month, then 3 months, then 6, 12, and then yearly.	Every 2-3 months	3 month intervals for the first year	Every 3 months

## Discussion

This survey observed strong agreement in terms of the treatment planning and treatment delivery aspects of spine radiosurgery. Especially safety and quality assurance of this novel treatment technique play a major role in the methods of all institutions. The following measures are considered as highly important for as safe-as-possible practice of spine radiosurgery. 1) Close cooperation between radiation oncologists and neurosurgeons especially in patients with epidural disease, spinal cord compression and instability; 2) Limitation of the target volume to maximum 3 vertebras; 3) Dedicated imaging protocols for target and organ-at-risk definition; 4) Anatomical target volume concepts with application of proper safety margins for the target volume and the spinal cord; 5) Highly conformal treatment planning, daily image-guidance, thorough patient immobilization and intra-fraction patient monitoring; 6) Close follow-up with repeated clinical and radiological response evaluation.

In contrast, three major areas of uncertainty and disagreement were identified: 1) Indications and contra-indications for spine radiosurgery; 2) treatment dose and 3) tolerance dose of the spinal cord. These areas will be discussed more in detail.

### Discussion with respect to Indication

#### Patient factors influencing indication for spine radiosurgery

Several prognostic scoring systems have been developed for overall survival after conventional, palliative radiotherapy for painful spine metastases [8,10,22] or metastatic spinal cord compression [23]. Recently, a recursive partitioning analysis (RPA) was reported for survival specific to spine SBRT [24]. The most favorable patients (Class 1, median OS of 21.1 months) were those with a time from primary diagnosis (TPD) > 30 months and a Karnofsky performance status (KPS) > 70, Class 2 was defined as those with a TPD > 30 months and KPS < 70 or a TPD < 30 months and age < 70 years (median OS of 8.7 months), and Class 3 was associated with the poorest outcomes and defined as TPD < 30 months and age > 70 years (median OS of 2.4 months). One could argue that spine radiosurgery would seem well indicated in patients with a longer life expectancy given that pain response of conventional doses is typically limited to 3 - 6 months (median duration of pain response) and multiple studies reported excellent pain control for a duration of 12 months after radiosurgery [12,13,25-27].

On the other hand, patients with short life expectancy could also benefit from spine radiosurgery; however, the rationale for radiosurgery would then be achievement of more rapid pain relief. For conventional radiotherapy, a mean pain response time of 3 weeks was reported by Van Der Linden et al. [28]. Ryu et al. and Chang et al. reported a slightly shorter median time to maximum pain relief of about 2 weeks such that even RPA class 3 patients could benefit from spine radiosurgery [12,25]. Whether this difference in pain relief reaches statistical significance needs to be demonstrated by a prospective trial. Until then, spine radiosurgery should be considered as a viable if not preferable option when rapid pain relief is required.

#### Tumor factors influencing indication for spine radiosurgery

Radiosurgical treatment of patients with epidural involvement is a major point of controversy. For tree institutions, epidural involvement was not a relevant factor in the decision making process for spine radiosurgery, whereas two institutions either required a clearance margin between the GTV and the spinal cord or preferred a surgical procedure prior to radiosurgery. Of note, the RTOG 0631 trial mandates a margin of 5 mm between the GTV and the spinal cord. This variation in indication is most likely explained by the risk of recurrence at the epidural space: three studies reported that 47 - 50% of all local failures after spine radiosurgery occur at the epidural space [12,27,29]. Sahgal et al. reported a trend towards local tumor recurrence if the GTV was within a distance of ≤1 mm to the spinal cord [30] and the minimum dose delivered to the target volume was shown as correlated with local tumor control [31].

A strategy to cope with epidural disease could be the application of fractionation schemes, which are adapted to the extent of epidural involvement [32]: single-fraction radiosurgery for targets with a clearance margin between the tumor and the spinal cord and fractionated radiosurgery if this clearance margin is violated. This fractionation could make use of basic radiobiology and deliver higher biological effective doses to the epidural tumor. However, the details regarding clearance margin and number of treatment fractions still have to be defined.

### Dose-fractionation

As previously mentioned, no dose response has been established for conventional palliative radiotherapy with doses between 8 Gy in 1 fraction and 40 Gy in 20 fractions. One explanation could be that all tested doses are well below established thresholds from radical radiotherapy: e.g. even the "high dose" approach of 40 Gy in 20 fractions would result in tumor control of less than 5% for NSCLC [33]. This hypothesis is supported by three studies, which reported a significant dose response relationship for primary [17,31] and re-irradiation [34] spine radiosurgery. The Memorial Sloan-Kettering Cancer Center (MSMCC) group reported excellent local control of > 95% for a prescription dose of 24 Gy and a minimum dose of > 15.1 Gy; however, the results are based on a retrospective review of serially dose-escalated patients and require validation before conclusions can be drawn. For pain control as primary endpoint, Ryu et al. reported a lower prescription dose threshold of 14 Gy; this dose response was not statistically significant [25]. It is important to interpret all these treatment doses in the context of the target and OAR volumes, which were used in the specific trials. Clinical application of any dose specification without detailed knowledge and application of the respective target and OAR concept cannot be recommended.

Centers in this study use a large rage of fractionations between single fraction to 10 fractions. Moreover, even within the single fraction treatment, the applied doses range between 16 Gy to 24 Gy. In the US, radiosurgery and stereotactic body radiotherapy are defined as treatment with maximum five fractions. In this study, one institution uses a 10 fraction approach, which does not fall under this definition. Nevertheless, we use the term radiosurgery even for this 10 fraction regimen because of two reasons: 1) the clinical and technical practice of the 10 fraction regimen is identical to the regimens using 1- 5 fractions; 2) using the LQ model, the biological effective dose of the 10 fraction regimen is expected to be at least equivalent to the 1- 5 fractions regimens. However, the limitations of the LQ model need to be considered for very high single fraction doses [35].

### Tolerance dose of the spinal cord

The third area of uncertainty and disagreement is the tolerance dose of the spinal cord. There was agreement between 4/5 institutions that a planning organ-at-risk should be generated with a safety margin around the true spinal cord. However, this safety margin varied between 1 mm around the spinal cord to 2 mm around the spinal canal. The dosimteric parameter used as dose threshold varied between D_max_, D10, D_0.1 cc _and D_2 cc_. The closest agreement was observed for single fraction radiosurgery, where dose thresholds of 10 - 11 Gy are used.

A very low incidence of myelopathy after spine radiosurgery has been described in the literature, despite dose escalation and hypo-fractionation. Ryu et al. observed a myelopathy in 1 out of 86 patients with a minimum follow-up of one year; these authors recommended a tolerance dose of 10 Gy as D10 [36]. Combined data from Stanford University Medical Center and University of Pittsburgh Medical Center reported an incidence of 5 out of 1075 patients, and it was recommended limiting the volume of spinal cord treated above an 8-Gy equivalent dose [37]. Sahgal et al. collected five cases of myelopathy and compared various dose volume parameters with 29 patients, who did not develop myelopathy [38]; all patients were treated with various radiosurgical fractionations. The thecal sack was delineated as the planning organ-at-risk volume for all cases and controls with centralized review of the DVH data. Doses were converted to 2 Gy-equivalent doses based on an α/β = 2 Gy to cope with variation of fractionation. The maximum point volume EQD_2 Gy _within the thecal sac significantly correlated with the risk of myelopathy as opposed to the larger volumes investigated (0.1 cc, 2 cc, and 5 cc). An EQD_2 Gy _threshold of 30 - 35 Gy was recommended for 1 - 5 fractions. In conventionally fractionated radiotherapy, the tolerance of the spinal cord is usually accepted between 45 - 50 Gy, despite that even a dose of 60 Gy results in a risk of myelopathy of only approximately 5% [39]. This disagreement between EQD_2 Gy _thresholds based on hypo-fractionation and thresholds based on conventionally fractionated radiotherapy has recently been confirmed by Daly et al. [40]. Further research is consequently required in this field as we do not yet understand the biologic ramifications of high dose per fraction on the normal tissues or tumor itself.

## Conclusions

Strong agreement in the methods of spine radiosurgery was observed. In particular, similarities were observed with safety and quality assurance playing an important role in the methods of all institutions: cooperation between neurosurgeons and radiation oncologists in case selection, dedicated imaging for target- and organ-at-risk delineation, application of proper safety margins for the target volume and organs-at-risk, conformal planning and precise image-guided treatment delivery, and close clinical and radiological follow-up. In contrast, three major areas of uncertainty and disagreement were identified: 1) Indications and contra-indications for spine radiosurgery; 2) treatment dose and fractionation and 3) tolerance dose of the spinal cord. Further research in form of retrospective and especially prospective studies is required to refine spinal radiosurgery in terms of safety and efficacy.

## Competing interests

This research was partially supported through an Elekta research grant with all institutions being members of the Elekta Spine Radiosurgery Research Consortium. This work and these data, however, are the intellectual property of the individual group members and their sponsoring institutions.

## Authors' contributions

MG designed the study, collected the data and performed the data analysis. RAS participated in data collection and analysis. JCF, PDG, RK, JS, AS participated in data collection. All authors performed critical review of the manuscript and finally approved the manuscript.
